# An overview of motor unit number index reproducibility in amyotrophic lateral sclerosis

**Published:** 2019-07-06

**Authors:** Davood Fathi, Shahriar Nafissi, Shahram Attarian, Christoph Neuwirth, Farzad Fatehi

**Affiliations:** 1 Shariati Hospital, Tehran University of Medical Sciences, Tehran, Iran; 2 Brain and Spinal Cord Injury Research Center, Neuroscience Institute, Tehran University of Medical Sciences, Tehran, Iran; 3 Reference Centre for Neuromuscular Disorders and ALS, CHU La Timone, Aix-Marseille University, Marseille, France; 4 Neuromuscular Disease Unit/ALS Clinic, Kantonspital St. Gallen, St. Gallen, Switzerland; 5 Iranian Center of Neurological Research, Tehran University of Medical Sciences, Tehran, Iran

**Keywords:** Motor Unit Number Index, Amyotrophic Lateral Sclerosis, Reproducibility of Results

## Abstract

Motor unit number index (MUNIX) is an electrophysiological technique to give an estimate of functioning motor neurons in a muscle. For any given neurophysiological technique for the use in clinical or research studies, reproducibility between different operators and in a single operator in different times is one of the most important qualities, which must be evaluated and approved by different examiners and centers. After its introduction, testing the reproducibility of MUNIX was the aim of many studies to show this quality of the technique. In this review, we aimed to summarize all the studies, which have been performed up to now to approve MUNIX reproducibility in amyotrophic lateral sclerosis comparing healthy individuals.

## Introduction

Progressive degeneration of motor neurons is the leading pathophysiologic characteristic of amyotrophic lateral sclerosis (ALS) in addition to upper motor neuron involvement. Progression of ALS could be monitored by clinical measures such as Medical Research Council (MRC) Scale for Muscle Strength, the revised ALS Functional Rating Scale (ALSFRS-R), and the older electrophysiological techniques including nerve conduction studies (NCSs) and needle electromyography (EMG). None of these measures has the potential of quantification of lower motor neuron degeneration in ALS.^1^^-^^4^ As in the last decades, there has been increasing number of clinical trials attempting to find an effective treatment for ALS, the introduction of a clinical or electrophysiological marker with the ability of quantitative evaluation of motor neuron loss was an essential need. The first try to make a numerical estimate of motor neurons started by McComas et al. with motor unit number estimation (MUNE).^   5 ^

After the original method of incremental stimulation MUNE introduced by McComas et al.,^   5 ^ other newer methods have been developed including multiple point stimulation (MPS),^   6 ^ statistical,^   7 ^ and spike-triggered averaging (STA) MUNE techniques.^     8 ^ MUNE value is calculated as supramaximal compound muscle action potential (CMAP) amplitude or area divided by the average size of surface-recorded motor unit potentials (SMUP) amplitude or area.^2^^-^^4^ The main electrophysiological advantage of MUNE is the potential to overcome the effect of reinnervation process occurring in the setting of chronic denervation such as ALS, which leads to maintaining the CMAP amplitude in the normal range despite the loss of more than 50% of motor axons.^9^^-^^11^ In addition, it can quantitatively measure the number of functioning motor neurons.^   12 ^ Different MUNE methods are usually time-consuming and are practically difficult to use in the everyday setting of managing patients and also in clinical trials. Considering these drawbacks, a new MUNE technique has been developed by Nandedkar et al. using surface EMG interference patterns.^13^^,^^14^ Unlike the other MUNE techniques, motor unit number index (MUNIX) does not need too many electrical stimulations and is fast and easy to perform in clinical practice.^12^^,^^15^ 

Recently, in another paper, we did a systematic review on MUNIX application, mainly focusing on different aspects including the reproducibility of the technique.^   16 ^ As the reproducibility of a given test is a critical issue in evaluating its power and applicability especially in disease conditions, the present review tries to gather all MUNIX studies in ALS up to now with unique insight into the technique and all attempts to approve its reproducibility in comparison with available data in healthy subjects. 

**Search strategy**

We accomplished a systematic search in English medical literature published in two databases including PubMed and SCOPUS for articles that comprised the keywords “motor unit number index” or “MUNIX” and “Amyotrophic Lateral Sclerosis”, “Motor Neuron Disorder” or “ALS”. All article types including cohort, case-control cohorts, case series, and case reports were included. The abstracts of all recruited articles were reviewed by two reviewers (DF and FF), and manuscripts containing points about reliability and reproducibility of MUNIX in ALS were included in this review.

**Technique of MUNIX**

MUNIX procedure was introduced initially by Nandedkar et al.^13^^,^^14^ The skin temperature must be maintained above 32 ^º^C. It is accomplished in three steps. First, a supramaximal CMAP is obtained in a standard tendon-belly setting of surface electrodes with optimizing the R1 electrode position to record the highest available CMAP amplitude. Obtaining a suboptimal CMAP amplitude will cause a false decrease in MUNIX values. For computing amplitude, area, and power of the CAMP curve, the negative phase is selected.

In the second stage, the surface interference pattern (SIP) is recorded. SIP is recorded by asking the patient to produce five or more distinct force levels [about 10% (or less), 25%, 50%, 75% (submaximal), and 100% of maximal force] for a few seconds each and the associated SIP is recorded for 300 milliseconds (ms). With repeating the latter process, we will have 10 or more SIPs. The stability of the surface EMG pattern at distinct force levels could be controlled by auditory and visual feedback.^9^^,^^14^^,^^17^ For setting the band-pass filter, it is recommended to use a filter pass range of 3 to 3000 Hz for both CMAP and SIP recording.^   14 ^

**Figure 1:**
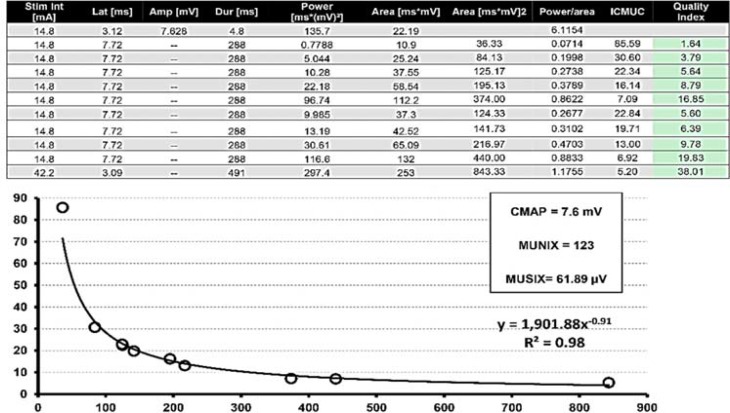
The above figure shows the detail of calculating motor unit number index (MUNIX) and motor unit size index (MUSIX) in right abductor pollicis brevis (APB) of a healthy person. In the bottom table, the raw data of compound muscle action potential (CMAP) and 10 surface interference patterns (SIPs) in terms of area and power is used to calculate ideal case motor unit count (ICMUC) with the quality indexes in the accepted (green) range. In the left upper part, CMAP amplitude and MUNIX and MUSIX values are depicted. In the right upper part, regression analysis curve with R^2 ^= 0.98 is shown. This is an example from Keypoint.NET Systems, when the calculation has to be performed manually without direct calculation within the electromyography (EMG) system.

At the final step, area and power values of CMAP and area and power values of 10 SIPs or more - depending on the used EMG system and software - are transferred to a Microsoft windows-based formula to determine MUNIX and motor unit size index (MUSIX) (Figure 1). Newer software systems offer a direct calculation embedded to the EMG system. For this calculation, raw data of CMAP and SIP are used to calculate the ideal case motor unit count (ICMUC), which is the motor unit number in a theoretical model supposing that all motor unit potentials are identical and not superimposed.^9^^,^^14^^,^^17^ 

ICMUC is plotted against the SIP area assuming SIP area as an index of force and ICMUC is calculated by this formula: ICMUC = A (SIP area)^α^, in which A and α are calculated by regression analysis. The following formula calculates MUNIX: MUNIX = A (20)^α^. In this formula, 20 is assumed as SIP area of very slight activity which could produce a SIP area of around 20 mV/ms. By using the assumptions of A and α, Nandedkar et al. could compare the ICMUC value among different subjects and muscles.^13^^,^^14^

For calculating MUSIX in microvolt (µV), CMAP amplitude is divided by MUNIX according to the following formula: [CMAP amplitude/MUNIX * 1000].^9^^,^^14^ MUSIX is the mean amplitude of SMUP. Unlike other MUNE techniques, in MUNIX, firstly MUNE is calculated and then the SMUP amplitude, whereas the route for calculation in other MUNE technique is vice versa.^14^ In comparison with the previous MUNE techniques, MUNIX is easy to perform for the examiner and to cooperate with the patient. The only drawback in performing MUNIX is the need for patient’s cooperation, as the investigator should ask the patient to increase force incrementally, while we can do other techniques of MUNE also in uncooperative and unconscious patients or animals.

It is noticeable that force direction may affect MUNIX value in multifunctional muscles.^18^^,^^19^ The effect of force direction in first dorsal interosseous (FDI) muscle on MUNIX value in abduction versus flexion was investigated and it was found that the average MUNIX for FDI flexion was slightly higher than that of FDI abduction in healthy individuals and a similar pattern in patients with ALS but in a lower range than the healthy people’s MUNIX values was found.^18^^,^^19^ According to these studies, for having a valid comparison in clinical practice and research studies, the precise direction of force in a given muscle for performing MUNIX should be considered in the study protocol.

Kaya et al. proposed another refinement in the technique of MUNIX, as they tried to control the contraction level by providing visual feedback on a screen for the healthy individuals who were investigated for MUNIX to a better adjustment of exerted force about the desired force level. They tried to standardize the technique to prevent potential errors, which could be inferred by the subjective misperception of contraction level.^20^

Regarding the quality of technique, it is recommended to perform MUNIX in muscles with the CMAP amplitude of at least more than 0.5 mV, as in less than this amplitude the reproducibility of technique falls below the acceptable levels.^   14 ^ In the latest guideline introduced by the developers of the method, they recommended positioning of active (R1) electrode to have the highest possible amplitude of CMAP and using at least twenty SIPs for 500 ms for optimizing the technique.^   21 ^

**Validation of MUNIX reproducibility**

Like any other new method in electrophysiology, MUNIX after introduction passed a series of investigations in different centers by different users to be accepted as a reliable technique in terms of intra-rater and inter-rater application. All studies related to the reproducibility of MUNIX in ALS or healthy people are summarized in table 1.

**Table 1:** Reproducibility of motor unit number index (MUNIX) in different studies

**Study author(s)**	**Subjects**	**Methods**	**Results**
Ahn et al. ^17^	62 controls	MUNIX in ADM	Inter- and intra-rater CC vs. CoV = 0.74 and 0.86 vs. 17.5% and 15.3%, respectively.
	22 ALS		inter- and intra-rater CC vs. CoV = 0.95 and 0.93 vs. 23.7% and 24.0%, respectively
Nandedkar et al. ^14^	10 controls	MUNIX in ADM	Intra-rater ICC = 0.64 and CoV = 16.8%
Neuwirth et al. ^11^	66 controls	MUNIX in 5 to 6 muscles	Highest intra-rater ICC was for AH and EDB = 0.83 and 0.81, respectively
			highest inter-rater ICC was for AH and ADM = 0.69 (for both)
			highest variability was for APB (ICC = 0.52)
Nandedkar et al. ^25^	19 ALS	MUNIX in 18 APB and 18 ADM	Inter-rater correlation and ICC > 0.90 and > 0.89, respectively
Kaya et al. ^20^	19 controls	MUNIX in APB	ICC = 0.76, CoV = 17.5%
Ahn et al. ^26^	41 controls	MUNIX in Orbicularis oculi	CC = 0.80 (Pearson), CoV = 20.9%
Neuwirth et al. ^22^	51 ALS	MUNIX in APB, ADM, BB, TA, EDB, and AHB	Intra-rater ICC = 0.89, inter-rater = 0.80 for mean MUNIX at 12-month follow-up
			intra-rater and inter-rater ICC for mean MUNIX = 0.87 and 0.84, respectively
Neuwirth et al. ^23^	50 controls	MUNIX in nasalis muscle	Intra-rater ICC = 0.87 for healthy controls and 0.92 for patients with ALS
	20 bulbar ALS		Inter-rater ICC for combined ALS/healthy group (n = 14): 0.97
Fathi et al. ^27^	30 controls	MUNIX in APB and TA bilaterally	CC ≥ 0.66, ICC ≥ 0.80
	30 ALS		CoV = 11.68% to 24.93%
			Baseline CC ≥ 0.87, follow-up CC ≥ 0.89, baseline ICC ≥ 0.93, follow-up ICC ≥ 0.94, baseline CoV = 13.98% to 25.98%, follow- up CoV = 13.90% to 32.95%
Neuwirth et al.^28^	Single volunteer	MUNIX in APB, ADM, BB, TA, EDB, and AHB by 12 examiners in a “round robin” set up on consecutive days	Mean intra-rater CoV = 14.0%, mean inter- rater CoV = 18.1%
Gawel and Kuzma- Kozakiewicz ^24^	15 ALS	MUNIX in APB, ADM, BB, TA, EDB, and AHB	No significant difference between MUNIX of test and re-test measurements in all six tested muscles (P > 0.05)
Escorcio-Bezerra et al. ^29^	51 controls	MUNIX in APB, ADM, and TA	Intra-rater ICC ≥ 0.71
			CoV ≤ 15.7%
	30 ALS		Intra-rater ICC ≥ 0.81
			CoV ≤ 23.7%
Escorcio-Bezerra et al. ^30^	21 controls	S-MUNIX and mean of three measurements of MUNIX (M- MUNIX) at baseline and 3 months later as retest in APB, ADM, and TA	ICC for M-MUNIX vs. S-MUNIX for TA, 0.90 vs. 0.80; for APB, 0.98 vs. 0.81; and for ADM, 0.90 vs. 0.70, respectively
			CoV of M-MUNIX vs. S-MUNIX for TA, 9% vs. 13%; for APB, 3.1% vs. 5.4%; for ADM, 4.4% vs. 6.9%, respectively
Neuwirth et al. ^31^	27 centers with 36 raters	MUNIX of 6 muscles (APB, ADM, FDI, BB, TA, and EDB) two times in 4 subjects, CoV of all measurements had to be < 20%	Mean CoV of all raters at the first measurement: 12.9% ± 13.5%
			BB and FDI disclosed the highest repetition rates

MUNIX: Motor unit number index; ALS: Amyotrophic lateral sclerosis; ADM: Abductor digiti minimi; APB: Abductor pollicis brevis; BB: Biceps brachii; TA: Tibialis anterior; EDB: Extensor digitorum brevis; AHB: Abductor hallucis brevis; CC: Correlation coefficient; ICC: Intraclass correlation coefficient; CoV: Coefficient of variation; FDI: First dorsal interosseous; S-MUNIX: Single measurement of MUNIX; M-MUNIX: Mean of 3 MUNIX measurements

Ahn et al. evaluated the reproducibility of MUNIX on abductor digiti minimi (ADM) muscle in 62 healthy controls and 22 patients with ALS.^   17 ^ MUNIX had the inter- and intra-rater correlation coefficient (CC) of 0.74 and 0.86, respectively, in healthy controls, and 0.95 and 0.93 in patients with ALS, respectively (P < 0.01 in all). On the other hand, MUNIX showed an acceptable level of variability, expressed as coefficient of variation (CoV), as inter- and intra-rater CoV of 17.5% and 15.3%, respectively, in healthy controls, and 23.7% and 24.0%, respectively, in patients with ALS.^   17 ^

Nandedkar et al. investigated 10 healthy controls for reproducibility of the technique in ADM muscle. The intraclass correlation coefficient (ICC) for MUNIX of ADM muscle was 0.64, and the variation between test and retest was 16.8 calculated as Variation = 200 × Absolute value of MUNIX (test-retest)/(test + retest).^   14 ^

Neuwirth et al. investigated the reproducibility of MUNIX in a multicenter study (6 centers) in 66 healthy individuals in different muscles with the identical setting. Considering the effect of aging on motor neuron loss, they divided healthy subjects into two groups of between 20 to 59 years and 60 years or older. They showed a different level of reliability according to ICC among different centers and for different muscles. Taking into account that the center which introduced the technique and had several years of experience demonstrated higher levels of reproducibility (inter-rater ICC = 0.81 and intra-rater ICC = 0.90) for abductor pollicis brevis (APB) muscle which had the highest variability among the tested muscles, this study suggested that appropriate training could reach a higher and acceptable level of reproducibility. Among different tested muscles, abductor hallucis brevis (AHB) and extensor digitorum brevis (EDB) had the highest intra-rater reproducibility (ICC = 0.83 and ICC = 0.81, respectively), but AHB and ADM showed the most significant values of inter-rater reliability (ICC for both = 0.69). The most challenging muscle with the highest variability (ICC = 0.52) was APB mainly due to the differences in CMAP amplitude.^   11 ^

For testing inter-rater reproducibility of MUNIX in patients with ALS, Nandedkar et al. investigated APB and ADM muscles in the stronger hand of 19 patients with ALS. They found a strong correlation and high reproducibility between two operator’s MUNIX measurements with the figures of r > 0.9 and ICC > 0.89, respectively. Looking at the individual patient’s data, they noticed that calculation of CoV might exaggerate the extent of variation between two measurements when MUNIX was very low which was seen in the weak muscles. They showed that variability of MUNIX and CMAP was higher in APB than ADM caused by variability of CMAP measurements in APB, making ADM a better choice for follow-up studies in comparison with APB.^   25 ^

Kaya et al. investigated the reliability of MUNIX in ABP of young healthy controls in two separate visits by a 4-week interval using a modified technique as already mentioned. They showed an acceptable level of reliability (ICC = 0.76) and variability (CoV = 17.5%) for MUNIX in APB muscle in healthy individuals.^   20 ^

Ahn et al. in another study, for the first time, tried to show reproducibility and applicability of MUNIX in orbicularis oculi muscle in 41 healthy volunteers. CC and CoV for MUNIX were 0.80 and 20.9%, respectively. According to this study, a standard range for MUNIX in the orbicularis oculi muscles of healthy subjects is > 22 (95^th^ percentile). They proposed that MUNIX of cranial muscles could be used successfully for the assessment of severity and progression of bulbar-onset ALS.^   26 ^

Neuwirth et al., in a longitudinal multicenter study (51 patients with ALS of three centers over a 15-month follow-up), tested the reproducibility of MUNIX at baseline in a set of six muscles [APB, ADM, biceps brachii (BB), tibialis anterior (TA), EDB, and AH] with the results in harmony with the previous studies showing the intra-operator and inter-operator ICC of 0.89 and 0.80 for mean MUNIX, respectively. After 12 months, the corresponding values of intra-operator and inter-operator ICC for mean MUNIX were 0.87 and 0.84, respectively. For the first time, this study showed the persistence of reproducibility of MUNIX during the course of ALS.^   22 ^

In a study by Neuwirth et al., the reliability of MUNIX in nasalis muscle as another cranial muscle was evaluated in 50 healthy individuals and 20 patients with bulbar type ALS. They showed that MUNIX was applicable and well tolerated in nasalis muscle. Intra-rater ICC for MUNIX was 0.87 and 0.92 for healthy controls and patients with ALS, respectively. Inter-rater ICC of MUNIX for combined ALS/healthy group (n = 14) was 0.97. While they found a good reproducibility of MUNIX in nasalis muscle in both groups, there was no significant difference of MUNIX between healthy controls and patients with ALS suggesting lack of ability of nasalis muscle MUNIX in detecting motor neuron loss in the bulbar type of ALS.^   23 ^

Intra-rater reproducibility of MUNIX in 30 healthy individuals and 30 patients with ALS was assessed by three statistical approaches in APB and TA muscles bilaterally at baseline and in the progression of the disease after 3-4 months in the ALS group by the first author of this review. We showed a significant correlation between the two measurements of MUNIX in all tested muscles at baseline (r ≥ 0.87, P < 0.01), at the follow-up visit (r ≥ 0.89, P < 0.01), and in healthy controls (r ≥ 0.66, P < 0.01). There was an acceptable statistically significant reproducibility of MUNIX in all measured muscles at baseline in patients with ALS (ICC ≥ 0.93, P < 0.01), in healthy controls (ICC ≥ 0.80, P < 0.01), and at a follow-up visit of patients with ALS (ICC ≥ 0.94, P < 0.01). The CoV of MUNIX was in the range of 13.98% to 25.98% at baseline, 11.68% to 24.93% in healthy controls, and 13.90% to 32.95% at the follow-up visit. This study confirmed the stability of reproducibility of MUNIX during the progression of ALS and the potential of MUNIX to track the deterioration of ALS both in clinical practice and in clinical trials.^   27 ^

For evaluating the inter-center changeability of MUNIX and performing a quality control study on this neurophysiologic method, Neuwirth et al. had a chance to run a “round robin” test on a single person during European Network for the Cure of ALS (ENCLAS) meeting in Dublin, Ireland, 2015. Twelve investigators (6 experienced, 6 less-experienced) performed MUNIX in six diverse muscles (APB, ADM, BB, TA, EDB, and AHB) two times in one single person on successive days. They showed that mean intra-rater CoV of MUNIX was 14.0% (± 6.4%) ranging from the lowest as 5.8% (for APB) to the highest as 30.3% (for EDB). Mean inter-rater CoV was 18.1% (± 5.4%) ranging from the lowest as 8.0% (for BB) to the highest as 31.7% (for AHB). There were no significant differences in variability between experienced and less-experienced investigators. This quality control study of MUNIX confirmed an acceptable level of variability in the range of ≤ 20% in intra- and inter-rater situations of a round robin setting with the exceptions of inter-rater CoV for CMAP and MUNIX of AHB and the intra-rater CoV of MUNIX in EDB.^   28 ^

Gawel and Kuzma-Kozakiewicz tested the intra-rater reproducibility of MUNIX in 15 patients with ALS in the first visit on the stronger side of these patients. They found that there was no significant difference between MUNIX of test and re-test measurements in all six (APB, ADM, BB, TA, EDB, and AHB) tested muscles (P > 0.05), confirming the reproducibility of the technique via an opposite method in comparison with the other studies showing a non-significant difference between test and re-test measurements values.^24^

Escorcio-Bezerra et al. planned to assess the intra-rater reproducibility of MUNIX in 51 healthy controls and 30 patients with ALS.^29^ While MUNIX showed a good level of intra-rater reproducibility in three tested muscles (APB, ADM, and TA) in healthy individuals (ICC ≥ 0.83, ICC ≥ 0.71, and ICC ≥ 0.81, respectively) and patients with ALS (ICC ≥ 0.89, ICC ≥ 0.81, and ICC ≥ 0.93, respectively), variability of MUNIX presented as CoV was more in the patients with ALS in comparison with the healthy controls, which was in the same line as previous studies.^14^^,^^17^ 

Escorcio-Bezerra et al. in another study aimed to advance the reproducibility of MUNIX by comparing the reproducibility of MUNIX calculated by first measurement of MUNIX at baseline and three months later [single MUNIX (S-MUNIX)] in TA, APB, and ADM.^30^ The reproducibility of MUNIX was calculated by the mean of three measurements of the same order of muscles at baseline and after 3 months (M-MUNIX) in 21 healthy controls. They found that ICC figures calculated by M-MUNIX were higher than the one by S-MUNIX for each tested muscle (ICC for TA, 0.90 vs. 0.80; ICC for APB, 0.98 vs. 0.81; and ICC for ADM, 0.90 vs. 0.70, respectively). Looking at the CoV, they showed the same trend in favor of M-MUNIX in comparison with S-MUNIX (CoV for TA, 9.0% vs. 13.0%; CoV for APB, 3.1% vs. 5.4%; CoV for ADM, 4.4% vs. 6.9%, respectively). They concluded that M-MUNIX could be a better measure for tracking motor neuron loss in ALS owing to a better reproducibility. 

Finally, MUNIX entered in the real life setting by using as one of the endpoint measures in one drug clinical trial and another natural course study. Neuwirth et al. evaluated the preparing process of 36 examiners in 27 centers by asking them to pass a training course of MUNIX to have a CoV of less than 20% to be eligible to participate in the trials.^31^ There were substantial differences between centers and evaluators emphasizing the role of high-quality training of MUNIX technique to achieve an acceptable level of reproducibility for investigators in the clinical trials. Mean CoV of all examiners at the first measurements was roughly 13%, and the necessity for the repetitions to reach a CoV of all measurements to be below 20% (to pass the qualification process) ranged from 0 to 43 (mean of 10.7). BB and FDI muscles showed the highest repetition rates, outlining that training is an appropriate tool to reduce variability when comparing these results with those from other groups who performed this method without specific training.

## Conclusion

Reviewing all the studies related to the reproducibility of MUNIX in ALS in more than ten years after its introduction, has established that MUNIX is an easy to perform, fast, reliable, and reproducible electrophysiological index of motor neuron loss in both intra- and inter-rater manners in patients with ALS. The existing literature also approves that MUNIX is reproducible both in a cross-sectional setting and through the progression of ALS. This aspect is missing for most other electrophysiological methods. Having said all the aforementioned findings, it seems that it is time to start using MUNIX as a reliable routine outcome measure alongside other functional scales such as ALSFRS-R in clinical trials of new drugs for ALS.

## Notes:

**How to cite this article:** Fathi D, Nafissi Sh, Attarian Sh, Neuwirth C, Fatehi F. An overview of motor unit number index reproducibility in amyotrophic lateral sclerosis. Iran J Neurol 2019; 18(3): 119-26.
